# DNA methylome signatures of prenatal exposure to synthetic glucocorticoids in hippocampus and peripheral whole blood of female guinea pigs in early life

**DOI:** 10.1038/s41398-020-01186-6

**Published:** 2021-01-18

**Authors:** Aya Sasaki, Margaret E. Eng, Abigail H. Lee, Alisa Kostaki, Stephen G. Matthews

**Affiliations:** 1 Department of Physiology, University of Toronto, Toronto, ON Canada; 2 Lunenfeld-Tanenbaum Research Institute, Sinai Health System, Toronto, ON Canada; 3 Department of Obstetrics & Gynecology, Mount Sinai Hospital, University of Toronto, Toronto, ON Canada

**Keywords:** Predictive markers, Epigenetics and plasticity

## Abstract

Synthetic glucocorticoids (sGC) are administered to women at risk of preterm delivery, approximately 10% of all pregnancies. In animal models, offspring exposed to elevated glucocorticoids, either by administration of sGC or endogenous glucocorticoids as a result of maternal stress, show increased risk of developing behavioral, endocrine, and metabolic dysregulation. DNA methylation may play a critical role in long-lasting programming of gene regulation underlying these phenotypes. However, peripheral tissues such as blood are often the only accessible source of DNA for epigenetic analyses in humans. Here, we examined the hypothesis that prenatal sGC administration alters DNA methylation signatures in guinea pig offspring hippocampus and whole blood. We compared these signatures across the two tissue types to assess epigenetic biomarkers of common molecular pathways affected by sGC exposure. Guinea pigs were treated with sGC or saline in late gestation. Genome-wide modifications of DNA methylation were analyzed at single nucleotide resolution using reduced representation bisulfite sequencing in juvenile female offspring. Results indicate that there are tissue-specific as well as common methylation signatures of prenatal sGC exposure. Over 90% of the common methylation signatures associated with sGC exposure showed the same directionality of change in methylation. Among differentially methylated genes, 134 were modified in both hippocampus and blood, of which 61 showed methylation changes at identical CpG sites. Gene pathway analyses indicated that prenatal sGC exposure alters the methylation status of gene clusters involved in brain development. These data indicate concordance across tissues of epigenetic programming in response to alterations in glucocorticoid signaling.

## Introduction

Synthetic glucocorticoids (sGC) are administered to pregnant women at risk of preterm delivery, approximately 10% of all pregnancies. Antenatal exposure to sGC significantly reduces the risk of respiratory distress syndrome. However, when given in multiple courses, it is associated with reduced head circumference at birth^1^ and neurodevelopmental impairment in young children^2^. Rodents, non-human primates, and sheep prenatally exposed to elevated glucocorticoids (either by sGC or maternal stress) show behavioral phenotypes associated with psychiatric disorders, such as anxiety and depression, and increased vulnerability to developing chronic disease during adulthood^3–5^. The broad range of phenotypes suggests a system-wide response to elevated glucocorticoids during early development.

Epigenetic mechanisms such as DNA methylation can maintain mitotically heritable differences in gene expression potential in the absence of DNA sequence variations. DNA methylation, a covalent modification of the DNA molecule, is involved in stable programming of gene regulation and in mediating long-term effects on genome function, behavioral and physical phenotypes in life. The established role of epigenetic mechanisms in complex disease and genomic imprinting has spurred interest in the role of DNA methylation, as a potential mechanism of developmental programming of health trajectories. The relative stability of DNA methylation modifications, in comparison to RNA, and its potential as an adaptive mechanism mediating adverse environmental exposures, has made them attractive candidates for biomarker discovery^6,7^.

A major obstacle to understanding the role of epigenetic modifications in disease, however, is the inherent tissue specificity of epigenetic regulation. There is substantial evidence of epigenetic modifications that are cell-type specific. In many cases, therefore, epigenetic information present in easily obtainable samples in humans may not provide insights into the epigenetic etiology in the primary tissue of interest. Indeed, in humans, peripheral tissues such as whole blood are often the only accessible source of DNA for epigenetic analyses. In addition, the impact of environmental factors playing causal roles in disease etiology are often not known. An important question concerns whether DNA methylation modifications in blood are informative of changes in the brain methylome associated with prenatal exposure to elevated glucocorticoids.

We examined here in parallel genome-wide methylation profiles from the hippocampus and whole blood of juvenile guinea pig females subjected to maternal exposure to sGC using reduced representation bisulfite sequencing (RRBS). Guinea pigs share similar patterns of brain development with humans, and the primary glucocorticoid in guinea pigs is cortisol, as in humans^8^. We hypothesized that prenatal sGC administration would alter DNA methylation modifications in offspring hippocampus and whole blood. We compared these two tissue types to assess epigenetic biomarkers potentially indicative of common underlying molecular pathways affected by sGC exposure. There is strong evidence of a crosstalk between the immune system and the brain, and common molecular pathways in both tissues are known targets of glucocorticoids^9,10^. Here, we provide evidence that the impact of the maternal sGC exposure on DNA methylation is genome-wide. Importantly, we identify concordant methylation alterations in a subset of genes in the hippocampus and whole blood. These findings suggest that whole blood may constitute an informative proxy for brain methylation alterations associated with prenatal sGC exposure, and thus an important source of accessible tissue for biomarker discovery and development.

## Materials and methods

### Animals and tissue samples

Female guinea pigs (Hartley strain, Charles River Canada, St Constant, Quebec, Canada) were housed and bred in our animal facility as previously described^11^. Pregnant guinea pigs were subcutaneously injected with three courses of 1 mg/kg betamethasone (sGC; phosphate-acetate mix; Betaject, Sabex, Boucherville, Quebec, Canada) on gestational day (GD) 40/41, 50/51, and 60/61 or saline (Control) and gave birth at term (~GD67). To mimic the dose of sGC given to pregnant women in the management of preterm labor (~0.25 mg/kg), a 4-fold higher dose was administered in the guinea pig (1 mg/kg), as the glucocorticoid receptor (GR) in guinea pig has a 4-fold lower affinity for sGC than the human GR^12,13^. Postnatal day (PND) 14 corresponds to a peak phase of neurodevelopment, and one in which glucocorticoids have prominent effects on the function of specific brain regions including the hippocampus^14,15^. At PND 14, one female juvenile offspring per litter was randomly selected and euthanized by an unblinded experimenter. Female juvenile offspring were examined as they were recently shown to be more sensitive to prenatal sGC exposure than males^16^.

Animals were euthanized via isoflurane inhalation, and hippocampus and whole blood samples were immediately collected via cardiac puncture from each subject (1/litter, *n* = 6/tissue type/treatment: Suppl Fig. 1A). Sample sizes of *n* = 6/treatment have been used by our group to examine epigenetic and transcriptional changes associated with prenatal sGC exposure^16,17^. The samples were stored at −80 °C until processing. All procedures were performed according to protocols approved by the Local Animal Care Committee at the University of Toronto, in accordance with the Canadian Council for Animal Care.

### Reduced representation bisulfite sequencing

DNA from the hippocampus and whole blood of each animal was extracted using AllPrep Mini Kit (Qiagen: Cat#80204) and QIAamp DNA Mini Kit (Qiagen: Cat#51304), respectively. The extracted DNA was quantified with a Quant-iT PicoGreen dsDNA assay (ThermoFisher: Cat# P11496) and DNA quality was assessed by TapeStation at the Center for Applied Genomics in the Hospital for Sick Children, Toronto Canada. DNA Integrity Numbers (DINs) over 7 were used for further analysis. RRBS libraries were then generated from high-quality dsDNA (100 ng) for each subject using the RRBS Methyl-Seq System 1-16 (Ovation: Part# 0353) and EpiTect Fast DNA Bisulfite kit following the manufacturer’s protocols (Qiagen: Cat#59824). RRBS libraries were then sequenced by the Donnelly Sequencing Center at the University of Toronto (Toronto, Canada) on a NextSeq500 following the manufacturer’s protocols for single end reads of 75 bp read length. Samples were sequenced in multiplexes of 10, balanced by tissue type and treatment condition to limit potential batch effects.

### Differentially methylated CpG sites (DMCs) and blood-brain comparisons

Adapter sequences and low quality reads (*q* < 30) were trimmed using Trim Galore (https://www.bioinformatics.babraham.ac.uk/projects/trim_galore/) followed by additional filtering and trimming using a python script provided by NuGEN to remove reads that did not contain an MspI site signature at the 5′ end. The python script is available on GitHub (https://github.com/nugentechnologies/NuMetRRBS/blob/master/trimRRBSdiversityAdaptCustomers.py). The reads were then aligned using Bismark^18^ and sorted by Samtools^19^. We used MethPipe^20,21^ to covary batch and identify differentially methylated CpG sites with at least 10X reads, an FDR < 0.05 and with at least 5% methylation difference.

DNA methylation profiles were compared between offspring of mothers that were exposed to sGC compared to controls in hippocampal and blood samples collected from each subject. CompEpitool^22^ was used to annotate the DMCs to known genes in guinea pigs, based on Broad Institute cavPor3. The bisulfite conversion rate and the reproducibility of the data were calculated using the default setting in methylKit^23^. We used two strategies to identify methylation modifications associated with genes in common between the hippocampus and blood. First, we identified a list of 134 genes showing differential methylation in both tissue types. Second, we identified a subset of 61 genes containing the same CpG sites differentially methylated in both tissue types. This subset of 61 genes was used for analyses focused on site-specific DNA methylation modifications localized to genes. Correlational analyses on common DMCs between the hippocampus and whole blood were performed using Spearman correlations in R.

### Gene pathway enrichment

Gene pathways associated with differentially methylated genes were explored using the biological process category in Gene Ontology (GO). For this analysis, we examined corresponding genes differentially methylated in both the hippocampus and the blood regardless of the specific CpG site(s) differentially methylated in each tissue type. The enrichment analysis was performed using g:Profiler with cavia porcellus (Guinea Pig) as the reference organism^24^ with significant enrichment defined by an FDR < 0.05. The list of significant GO terms were then visualized and interpreted by using Cytoscape^25^. Cytoscape clusters main biological themes based on a list of significant GO terms that often include related nodes of shared biological pathways. This feature enabled the assignment of functional annotations to differentially methylated genes in common between hippocampus and blood.

### Enrichment of DNA motifs and transcription factor binding sites

To identify DNA motifs associated with transcription factor binding sites, sequence motifs were examined at differentially methylated CpG sites ±100 bp^26^. Then, MEME suite 5.0.4 was used to identify overrepresented sequences using the following parameters: -dna, -mod anr, -nmotifs 20, -revcomp, and -markov-order1. The output of MEME is a list of position weight matrix (PWM) visualized as sequencing logos, where the height of each nucleotide in the logo is proportional to its probability. We used a background model within MEME in order to normalize for biased distribution of high numbers of CpG nucleotides present in our sequences due to enrichment of regulatory elements obtained from RRBS libraries. Finally, TOMTOM^27^ from the MEME suite was used to identify whether glucocorticoid regulatory elements (GREs) were enriched in the discovered motifs. Also, discovered motifs were searched against known transcription factors using the JASPAR 2018 CORE (non-redundant) database for vertebrates^28^. *E-*values less than 0.05 are considered significant as a default of the program.

## Results

The bisulfite conversion rate was greater than 99% for all of the samples. Supplementary Fig. 1B, C provides a matrix of correlational coefficients of DNA methylation for each tissue type, showing pair-wise comparisons between all samples. We found that the correlation coefficients were greater than 0.8 for all pairwise comparisons, indicating high levels of reproducibility in our dataset^23,29^.

### Differentially methylated CpG sites (DMCs)

**Fig. 1:**
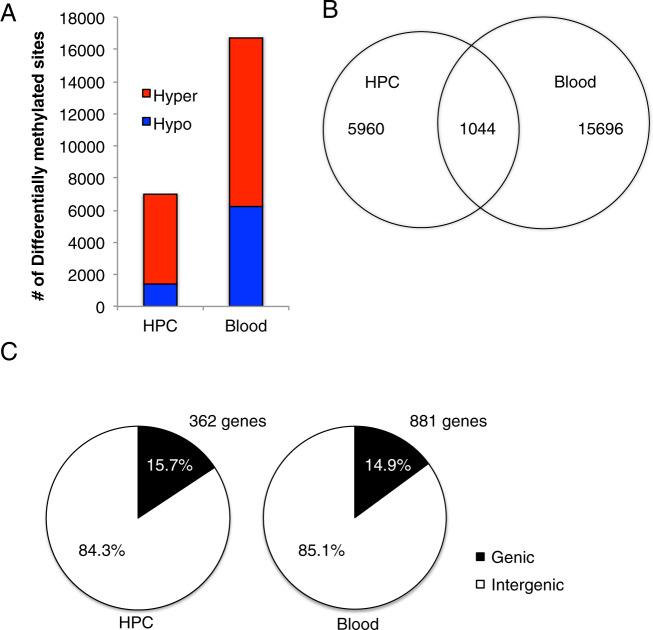
Overview of differentially methylated CpG (DMCs) in the hippocampus (HPC) and whole blood in female offspring born to mothers that received synthetic glucocorticoids (sGC) during pregnancy compared to saline controls. **A** Bar graphs illustrating the number of DMCs for the HPC and whole blood. Red bars: the number of DMCs that were hypermethylated in the sGC condition compared to controls. The blue bars: hypomethylation in the sGC condition compared to controls. **B** Venn diagram illustrating the number of the DMCs in each tissue and at the same CpG locations in both the HPC and blood. **C** Pie charts illustrating the proportion of genic (in black) vs intergenic (in white) DMCs in the HPC and whole blood. The number of genes associated with DMCs is shown for each tissue. DMCs were defined as significant at FDR *P* < 0.05 and 5% minimum methylation difference.

Overall, in the hippocampus we identified 7004 DMCs, of which 5581 or 79.9% were hypermethylated and 1423 or 20.3% were hypomethylated in response to sGC exposure (Fig. 1a). In the blood, we identified 16,740 DMCs of which 10,494 or 62.7% were hypermethylated and 6246 or 37.3% were hypomethylated in response to sGC exposure. There were 1044 DMCs localized to the same CpG sites in both the hippocampus and blood (Fig. 1b), representing 14.9% and 6.2% of the total number of DMCs identified in the hippocampus and blood, respectively. Supplementary Table 1A–C provide the full lists of DMCs identified in the hippocampus, blood and the DMCs at the same CpG locations between the two tissues, respectively. Among overlapping DMCs, the majority were located in intergenic regions of the guinea pig genome, consisting of 84.3% and 85.1% of the DMCs in the hippocampus and the blood, respectively (Fig. 1c).

### Correspondence between methylation changes in blood and brain

**Fig. 2:**
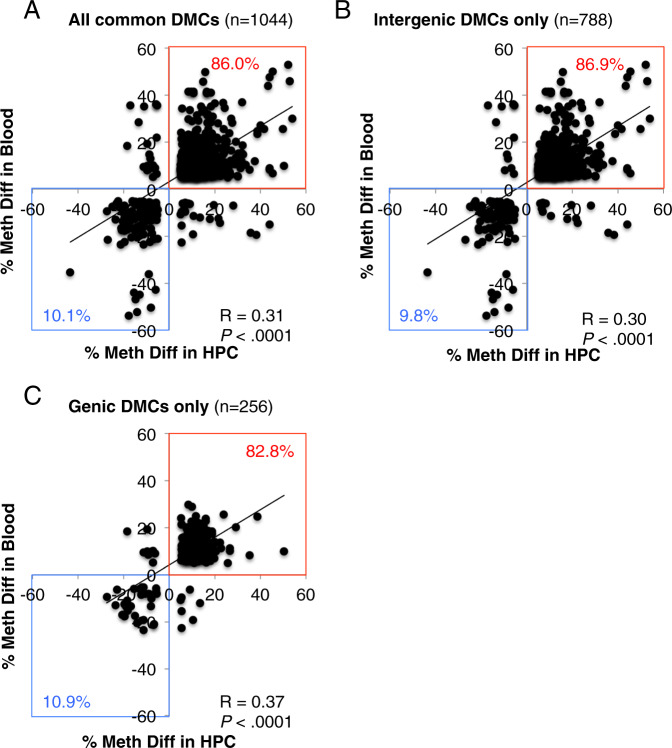
Methylation percent difference per CpG across the hippocampus (HPC) or whole blood in female offspring born to mothers that received sGC during pregnancy compared to saline controls. **A** Scatter plot for all DMCs in common between the two tissues (*n* = 1044). **B** Scatter plot for intergenic DMCs in common between the two tissues (*n* = 788). **C** Scatter plot for gene-associated DMCs in common between the two tissues (*n* = 256). The red highlighted areas indicate common DMCs that were hypermethylated in both tissues. The blue highlighted areas indicate common DMCs that were hypomethylated in both tissues. Correlation coefficients and *P*-values were determined by Spearman correlations. DMCs were defined as significant at FDR *P* < 0.05 and 5% minimum methylation difference.

To investigate the relationship between methylation changes associated with sGC exposure in the hippocampus and blood samples, correlation analysis was performed between the methylation difference percent among the 1044 DMCs in common between the hippocampus and blood. There was a positive correlation between the methylation difference percent in the hippocampus and the blood (*R* = 0.31, *n* = 1044, *P* < .0001; Fig. 2a). Likewise, this significant positive relationship held when the DMCs were further separated into intergenic DMCs only (788 DMCs; *R* = 0.3, *n* = 788, *P* < .0001; Fig. 2b) and genic DMCs only (256 DMCs; *R* = 0.37, *n* = 256, *P* < .0001; Fig. 2c). Additionally, in each of the above correlations, over 90% of the DMCs in common between the two tissues showed the same directionality of methylation change (i.e., either hypermethylation or hypomethylation) in response to sGC exposure. These results indicate that the majority of the common DMCs were concordant between the hippocampus and blood samples.

For DMCs associated with genes, DNA methylation modifications were largely tissue-specific, though a subset of genes showed methylation changes in both tissues. The DMCs identified in the hippocampus were associated with 362 genes, whereas the DMCs identified in the blood were associated with 881 genes (see Fig. 1c and Suppl Table 1A, B). We identified 134 genes containing DMCs in both the hippocampus and the blood. Among these, 61 genes showed DMCs at identical CpG sites in both tissues. Table 1 shows a list of 20 top genes containing DMCs in common between the hippocampus and blood, ranked-ordered by the highest of numbers of DMCs combined between the two tissues. Supplementary Table 1D shows the full list of genes containing DMCs in both the hippocampus and blood.

**Table 1:** List of 20 top genes that showed DMCs in common between the hippocampus (HPC) and whole blood samples, rank-ordered by the greatest number of DMCs combined between the two tissue types.

Gene	Sum of DMCs (Brain/Blood)	Identical DMCs	Meth Diff (%) HPC		Meth Diff (%) blood		Function
			Range	Avg	Range	Avg	
NFIA	81 (28/53)	22	−6.4 to 18.5	10.1	−11.9 to 29.6	10.7	Brain formation
CAMTA1	65 (39/26)	5	−11.1 to 27.5	12.1	6.0 to 24.7	11.4	Cognition
AGAP2	56 (14/42)	13	5.4 to 16.1	11.4	−22.7 to 23.8	9.3	Anti-apoptosis
PRDM16	46 (13/33)		−6.9 to 44.7	24.4	−10.3 to 16.4	8.2	Histone methyltransferase
MAP3K6	42 (18/24)	12	−8.8 to 26.4	11.8	−9.4 to 19.5	8.7	MAPK signaling pathway
SNX20	40 (8/32)	7	−5.3 to 50.4	14.2	5.2 to 37.5	19.2	Vesicle trafficking
PHLDB1	36 (12/24)	12	−9.6 to 18.8	9.9	5.6 to 19.1	9.5	Brain cancer
ROR1	34 (22/12)	3	−9.2 to 20.3	9.8	6.0 to 38.8	16.0	Neuronal growth
NAV1	33 (14/19)	14	−9.3 to 23.8	8.0	−7.9 to 25.6	9.1	Neuronal migration
MDGA1	30 (7/23)	4	5.2 to 17.9	10.8	−33.3 to 16.0	−8.3	Brain development
IRF2BPL	29 (8/21)	7	6.5 to 14.2	10.0	−13.7 to 19.4	9.6	Female reproduction
CASZ1	28 (9/19)	4	6.1 to 18.9	11.5	5.6 to 21.9	10.3	Vascular morphogenesis
ADGRD1	27 (4/23)		−9.7 to 10.8	4.5	−12.4 to 34.9	6.8	Adhesion G protein-coupled receptor
HM13	27 (14/13)	12	−19.4 to −7.0	−14.8	−21.3 to −5.9	−14.7	Immune system
KIF6	27(4/23)	3	6.1 to 17.8	12.4	−24.2 to 38.0	9.4	Heart function
PARVB	25(10/15)		−10.1 to 19.2	10.3	−40.1 to 10.8	−2.1	Neuronal migration
TNFRSF19	25 (12/13)	7	5.1 to 20.5	11.7	6.0 to 13.1	8.8	Apoptosis
KIF21B	24 (12/12)	12	7.7 to 16.0	12.0	12.2 to 28.8	20.3	Neuronal morpholology
NCLN	24 (20/4)	1	−26.5 to 18.8	2.2	−6.0 to 13.0	−0.9	Embryo development
SYN3	23 (1/22)		9.2	9.2	−14.5 to 17.3	3.0	Synaptogenesis

**Fig. 3:**
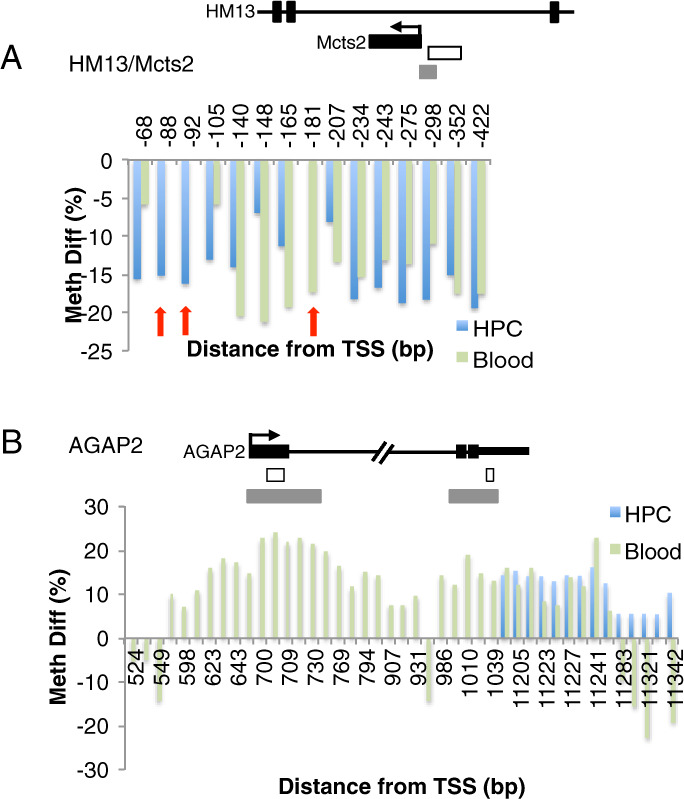
Examples of genes showing both differentially methylated regions (DMRs) that are similar across the hippocampus (HPC) and whole blood as well as tissue-type specific DMCs. The top of each panel shows the genomic region examined for **A** *HM13/Mcts2* and **B** *AGAP2*. The white horizontal boxes represent observed DMRs. The gray horizontal boxes represent CpG islands. In the bar graphs, the *x*-axis shows the distance in base pairs from the transcription start sites, while the *y*-axis shows the methylation percentage difference between sGC treatment and saline controls. Red arrows indicate tissue-type specific DMCs in *HM13/Mcts2*.

We found that multiple DMCs associated with a given gene were located in close proximity, forming differentially methylation regions (DMRs). For example, a CpG island in the intron region of *HM13* (Histocompatibility Minor 13) as well as in a promoter region of *MCTS2* (malignant T cell amplified sequence 2) was differentially hypomethylated with sGC exposure in both tissues, with the exception of a few tissue-specific DMCs (Fig. 3a). Interestingly, this CpG island is localized to an imprinted region, where DNA methylation is known to be laid down during early embryogenesis^30^. Similarly, in the *AGAP2* (ArfGAP With GTPase Domain, Ankyrin Repeat And PH Domain 2) gene, we observed that both tissue types showed an overlapping DMR in the CpG island located in the last exon but not in the CpG island in the first exon, where the DMR was specific to blood (Fig. 3b). Overall, we found that there were common CpG sites differentially methylated as a result of sGC exposure in both the hippocampus and whole blood, but with notable differences in the distribution of DMCs between the two tissue types.

### Gene pathway enrichment

**Fig. 4:**
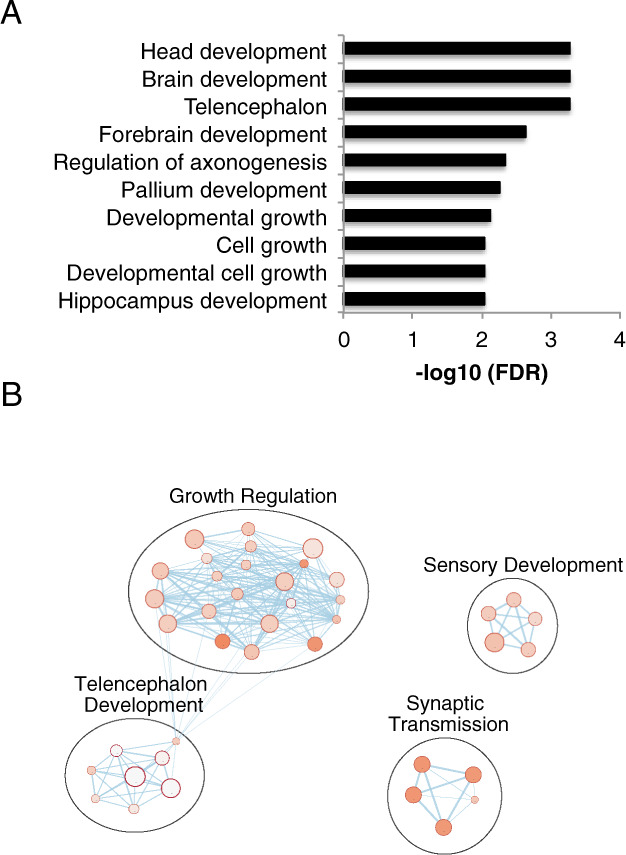
Gene ontology analysis of 134 genes whose methylation status was altered by prenatal sGC treatment in both the hippocampus and whole blood. **A** Top ten enriched GO terms identifies an enrichment for annotations associated with neurodevelopment. **B** Cytoscape image of enriched GO terms (57 terms) clustering based on shared gene pathways among terms. FDR *p*-values are color-coded in orange inside a node, with lighter nodes indicating lower FDR *p*-values). The size of each node represents the number of genes involved in each GO term, with edges indicating shared genes between annotations.

We performed Gene Ontology (GO) analysis to biologically contextualize genes found to be differentially methylated both in the hippocampus and blood. Gene set functional analysis using GO terms showed that the 134 genes in common were predominantly enriched in gene pathways involved in neurodevelopment. Figure 4a shows the top ten GO terms ranked by the lowest to highest FDR *p*-values, where seven of ten top GO terms involved neurodevelopment. The list of all significant GO terms was then visualized using Cytoscape^25^. Supplementary Table 2 provides the full list of GO terms for the differentially methylated genes in common between the hippocampus and blood. Analysis of related nodes of shared biological pathways revealed that the differentially methylated genes in common in the hippocampus and blood occurred in genes with shared functional roles in telencephalon development and growth regulation (Fig. 4b).

### Enrichment of DNA motifs and transcription factor binding site

We examined the DNA sequence motifs surrounding the differentially methylated CpG sites associated with genes to identify common regulatory elements. Synthetic glucocorticoids bind to glucocorticoid receptors, which act as transcription factors that affect the expression of many genes containing glucocorticoid regulatory elements (GREs). Differential DNA methylation can alter the accessibility of transcription factors to gene regulatory elements^31^. Our primary goal was to examine whether GREs were enriched in the sequences around the DMCs that were common in the hippocampus and blood. Among the 256 DMCs that were common in the hippocampus and blood and also associated with genes (see Fig. 2c), three significant DNA motifs were discovered. Among these motifs, two out of three motifs showed significant similarity to known transcription factors. One motif was similar to *EWSR1-FLI1* (EWS RNA binding protein 1-Fli-1 proto-oncogene, ETS transcription factor) and *ZNF263* (Zinc finger protein 263). Another motif was similar to *SP1* (Sp1 transcription factor), *SP2* (Sp2 transcription factor), and *ZNF263*. None of the DNA motifs sequences were similar to GREs.

We also examined DNA sequence motifs surrounding differentially methylated CpG sites associated with genes separately for the hippocampus and whole blood. In the hippocampus, 1556 DMCs were associated with genes. We identified two significant motifs: One motif was associated with *ZNF384*. Similar to the combined motif analysis for hippocampus and blood above, a second motif was associated with *SP2 and ZNF263*. In the whole blood, 3633 DMCs were associated with genes. We identified one significant motif. Similar to the result for the hippocampus alone, one motif was associated with *ZNF384*. As in the analysis of the common CpG sites in common between hippocampus and blood, we did not observe evidence for the enrichment of GREs.

## Discussion

Exposure to elevated glucocorticoids in pregnancy can have a long-lasting impact on offspring neurodevelopment^5^. Earlier work in animal models has largely focused on examining epigenetic differences in the brain, although the effects of developmental exposure to elevated glucocorticoids on offspring physiology are known to involve multiple tissue types^32^, including leukocytes. The current study was designed to examine evidence for common underlying molecular pathways showing DNA methylation modifications with sGC exposure in brain and blood. We addressed this question by comparing DNA methylation in young female guinea pigs that were prenatally exposed to sGC. Here, we identify for the first time loci in the hippocampus and whole blood that show similar epigenetic sensitivity to maternal sGC exposure.

Using a within-subject design, we find evidence for prominent tissue-specific differential methylation in this hippocampus and blood, as well as a small proportion of DMCs showing a significant correlation between both tissue types. These findings indicate that the majority of DMCs identified in this study were tissue-specific, at least in the regulatory elements examined using RRBS. Thus, researchers interested in the epigenetics of brain-related disorders should be careful when interpreting methylome-wide data assessed in peripheral tissues such as blood. However, at select loci, DNA methylation modifications in response to sGC exposure exhibited similarity between tissues, providing support for the interpretation that epigenetic dysregulation in tissues such as the hippocampus can be inferred from analyses of these loci in whole blood DNA. Most of the genes showing DNA methylation changes at identical CpG sites in both tissues displayed changes in methylation in the same direction (i.e., hyper-methylation or hypo-methylation at 58/61 genes). Several DMCs in common are associated with genes implicated in disease processes, including multiple sclerosis (*KIF21B*)^33^, schizophrenia and bipolar disorder (*MDGA1*)^33^ and autism spectrum disorder (*SORBS1*, *MEF2C*)^34,35^. Many of the genes in common also have known functions in brain development and cognition.

We noted the presence of DMRs at the same CpG sites in both tissues along with tissue-specific DMCs at some gene-associated loci, including *HM13/MCTS2*, an imprinted gene^36^. Importantly, these results suggest that the DMCs in the hippocampus were not merely a reflection of the DMCs in the blood, arguing against the possibility of an epigenetic signal driven by blood contamination in the brain. The role of DNA methylation in gene regulation is well documented in imprinted genes^37^. Many imprinted genes have been implicated in cognitive function and complex diseases in humans^38–40^. DNA methylation modifications at the *HM13/MCTS2* locus are laid down during gametogenesis and maintained throughout cell division^30,41^. As the typical pattern and timing of DNA methylation has been well documented at this locus^41^, our results indicate that sGC exposure during late pregnancy likely disrupted the methylation pattern of the *HM13/MCTS2* DMR. Interestingly, *MCTS2* is known to have been retrotransposed into intron 4 of *HM13/H13* in supraprimates only^30,42^, and is therefore unique to rodents and primates, including humans. Additional work is needed to examine the potential functional consequences of imprinting dysregulation at the *HM13/MCTS2* locus.

We performed in silico analysis to examine the enrichment of binding sites for specific transcription factors at differentially methylated sites. This analysis was performed to identify whether the DNA methylation differences we observed at multiple loci may be driven by common transcription factors (or vice versa). As such, we examined the sequence around the DMCs in common between tissue types, as well as the sequence surrounding DMCs present within each tissue. In both cases, we did not observe enrichment of GRE sequences, suggesting that the methylation difference overlap observed in the two tissues is unlikely to involve the direct molecular actions of sGC binding to GRE sites. Instead, our analysis suggests that the involvement of zinc finger transcription factors in both tissue types, notably ZNF263. ZNF263 has been implicated in a study of methylation differences in whole blood from children who exhibit elevated cortisol levels^43^. Specifically, chronic high levels of cortisol (measured in hair samples) were associated with a decrease in DNA methylation at ZNF263 binding sites across the genome. Little is known about the function of ZNF263, but it is conserved across mammalian species and thought to have a repressive effect on transcription^44^. Additional studies are needed to investigate the role of this transcription factor in regulating gene activity in response to alterations in cortisol.

Our findings raise several related questions for future study. First, what are the causal determinants of these epigenetic differences? Our results identifying differential methylation at the *HM13/MCTS2* DMR support recent emerging literature indicating that some imprinted genes develop persistent modifications in DNA methylation in response to environmental exposures, such as cell culture media content in in vitro fertilization settings and maternal nutrition^45^. Second, very little is known about the mechanisms by which exposure to maternal glucocorticoids during pregnancy affect the establishment of epigenotypes at specific loci. Contrary to our expectations, the DMCs loci were not enriched in DNA motifs for GRE binding sites of predicted glucocorticoid binding, according to our in silico analysis. It is likely that targeting of the methylation machinery involves other transcription factors, including members of the zinc-finger binding protein family. A third question concerns the functional relevance of DMCs localized to intergenic relative to genic regions. We found that the largest portion of concordant DMCs between two tissue types were located in intergenic regions, where recent advances in sequencing have implicated retrotransposable elements in the effects of stress on brain function^46,47^. Future studies using functional genomic assays are needed to address these questions. In addition, it will be an important to investigate these relationships in male offspring and how the relationships may change with age.

In conclusion, maternal exposure to sGC alters DNA methylation modifications at shared loci in the hippocampus and whole blood in a manner that persists into the juvenile phase. The present results support the growing evidence indicating crosstalk between the immune system and the brain, with the actions of glucocorticoids playing important roles in both tissues. The striking overlap between the changes in DNA methylation in the hippocampus and whole blood identified in this study supports the feasibility of assessing DNA methylation biomarkers of glucocorticoid exposure in utero among human populations. The sites of common methylation modifications may therefore represent candidate loci for future studies of human populations during early development, where brain tissue is not available. As such, the identification of common DNA methylation modifications in neural and peripheral tissues should advance translational studies of the impact of prenatal glucocorticoid exposure on epigenetics and human disease.

## Supplementary information

Suppl Fig Table Legends

Suppl Fig1

Suppl Table1A

Suppl Table1B

Suppl Table1C

Suppl Table1D

Suppl Table2

## Data Availability

The data discussed in this publication have been deposited in NCBI’s Gene Expression Omnibus^48^ and are accessible through GEO Series accession number GSE163447.

## References

[1] Murphy KE (2008). Multiple courses of antenatal corticosteroids for preterm birth (MACS): a randomised controlled trial. Lancet.

[2] Asztalos E (2014). Association between gestational age at birth, antenatal corticosteroids, and outcomes at 5 years: multiple courses of antenatal corticosteroids for preterm birth study at 5 years of age (MACS-5). BMC Pregnancy Childbirth.

[3] Moss TJ (2005). Effects into adulthood of single or repeated antenatal corticosteroids in sheep. Am. J. Obstet. Gynecol..

[4] Moisiadis VG, Matthews SG (2014). Glucocorticoids and fetal programming part 1: outcomes. Nat. Rev. Endocrinol..

[5] Matthews SG, McGowan PO (2019). Developmental programming of the HPA axis and related behaviours: epigenetic mechanisms. J. Endocrinol..

[6] Sasaki A, Kim B, Murphy KE, Matthews SG (2020). Impact of ex vivo sample handling on DNA methylation profiles in human cord blood and neonatal dried blood spots. Front. Genet..

[7] Horvath S (2012). Aging effects on DNA methylation modules in human brain and blood tissue. Genome Biol..

[8] Morrison JL (2018). Guinea pig models for translation of the developmental origins of health and disease hypothesis into the clinic. J. Physiol..

[9] Bellavance MA, Rivest S (2014). The HPA—immune axis and the immunomodulatory actions of glucocorticoids in the brain. Front. Immunol..

[10] Cain DW, Cidlowski JA (2017). Immune regulation by glucocorticoids. Nat. Rev. Immunol..

[11] Dean F, Matthews SG (1999). Maternal dexamethasone treatment in late gestation alters glucocorticoid and mineralocorticoid receptor mRNA in the fetal guinea pig brain. Brain Res..

[12] Owen D, Matthews SG (2007). Repeated maternal glucocorticoid treatment affects activity and hippocampal NMDA receptor expression in juvenile guinea pigs. J. Physiol..

[13] Keightley MC, Fuller PJ (1995). Cortisol resistance and the guinea pig glucocorticoid receptor. Steroids.

[14] Gould E, Tanapat P (1999). Stress and hippocampal neurogenesis. Biol. Psychiatry.

[15] Guidi S, Ciani E, Severi S, Contestabile A, Bartesaghi R (2005). Postnatal neurogenesis in the dentate gyrus of the guinea pig. Hippocampus.

[16] Moisiadis VG, Constantinof A, Kostaki A, Szyf M, Matthews SG (2017). Prenatal glucocorticoid exposure modifies endocrine function and behaviour for 3 generations following maternal and paternal transmission. Sci. Rep..

[17] Crudo A (2013). Effects of antenatal synthetic glucocorticoid on glucocorticoid receptor binding, DNA methylation, and genome-wide mRNA levels in the fetal male hippocampus. Endocrinology.

[18] Krueger F, Andrews SR (2011). Bismark: a flexible aligner and methylation caller for Bisulfite-Seq applications. Bioinformatics.

[19] Li H (2009). The Sequence Alignment/Map format and SAMtools. Bioinformatics.

[20] Dolzhenko E, Smith AD (2014). Using beta-binomial regression for high-precision differential methylation analysis in multifactor whole-genome bisulfite sequencing experiments. BMC Bioinform..

[21] Song Q (2013). A reference methylome database and analysis pipeline to facilitate integrative and comparative epigenomics. PLoS ONE.

[22] Kishore K (2015). methylPipe and compEpiTools: a suite of R packages for the integrative analysis of epigenomics data. BMC Bioinform..

[23] Akalin A (2012). methylKit: a comprehensive R package for the analysis of genome-wide DNA methylation profiles. Genome Biol..

[24] Reimand J (2016). g:Profiler-a web server for functional interpretation of gene lists (2016 update). Nucleic Acids Res..

[25] Reimand J (2019). Pathway enrichment analysis and visualization of omics data using g:Profiler, GSEA, Cytoscape and EnrichmentMap. Nat. Protoc..

[26] Oh G (2018). Cytosine modifications exhibit circadian oscillations that are involved in epigenetic diversity and aging. Nat. Commun..

[27] Gupta S, Stamatoyannopoulos JA, Bailey TL, Noble WS (2007). Quantifying similarity between motifs. Genome Biol..

[28] Khan A (2018). JASPAR 2018: update of the open-access database of transcription factor binding profiles and its web framework. Nucleic Acids Res..

[29] Bock C (2012). Analysing and interpreting DNA methylation data. Nat. Rev. Genet..

[30] Wood AJ (2007). A screen for retrotransposed imprinted genes reveals an association between X chromosome homology and maternal germ-line methylation. PLoS Genet..

[31] Schubeler D (2015). Function and information content of DNA methylation. Nature.

[32] Crudo A (2012). Prenatal synthetic glucocorticoid treatment changes DNA methylation states in male organ systems: multigenerational effects. Endocrinology.

[33] Goris A, Boonen S, D’Hooghe M, Dubois B (2010). B. Replication of KIF21B as a susceptibility locus for multiple sclerosis. J. Med. Genet..

[34] Nowakowska BA (2010). Severe mental retardation, seizures, and hypotonia due to deletions of MEF2C. Am. J. Med. Genet. Part B.

[35] Tu S (2017). NitroSynapsin therapy for a mouse MEF2C haploinsufficiency model of human autism. Nat. Commun..

[36] White CR, MacDonald WA, Mann MR (2016). Conservation of DNA methylation programming between mouse and human gametes and preimplantation embryos. Biol. Reprod..

[37] Li E, Beard C, Jaenisch R (1993). Role for DNA methylation in genomic imprinting. Nature.

[38] Nicholls RD (2000). The impact of genomic imprinting for neurobehavioral and developmental disorders. J. Clin. Investig..

[39] Wilkinson LS, Davies W, Isles AR (2007). Genomic imprinting effects on brain development and function. Nat. Rev. Neurosci..

[40] Tucci V, Isles AR, Kelsey G, Ferguson-Smith AC, Erice Imprinting, G (2019). Genomic imprinting and physiological processes in mammals. Cell.

[41] Wood AJ (2008). Regulation of alternative polyadenylation by genomic imprinting. Genes Dev..

[42] Suzuki S, Shaw G, Kaneko-Ishino T, Ishino F, Renfree MB (2011). The evolution of mammalian genomic imprinting was accompanied by the acquisition of novel CpG islands. Genome Biol. Evol..

[43] Natt D, Johansson I, Faresjo T, Ludvigsson J, Thorsell A (2015). High cortisol in 5-year-old children causes loss of DNA methylation in SINE retrotransposons: a possible role for ZNF263 in stress-related diseases. Clin. Epigenet..

[44] Frietze S, Lan X, Jin VX, Farnham PJ (2010). Genomic targets of the KRAB and SCAN domain-containing zinc finger protein 263. J. Biol. Chem..

[45] Waterland RA, Jirtle RL (2004). Early nutrition, epigenetic changes at transposons and imprinted genes, and enhanced susceptibility to adult chronic diseases. Nutrition.

[46] McEwen BS (2015). Mechanisms of stress in the brain. Nat. Neurosci..

[47] Hunter RG (2012). Acute stress and hippocampal histone H3 lysine 9 trimethylation, a retrotransposon silencing response. Proc. Natl Acad. Sci. USA.

[48] Edgar R, Domrachev M, Lash AE (2002). Gene Expression Omnibus: NCBI gene expression and hybridization array data repository. Nucleic Acids Res..

