# Supplementary material for: DNA methylome signatures of prenatal exposure to synthetic glucocorticoids in hippocampus and peripheral whole blood of female guinea pigs in early life
Source: Transl Psychiatry. 2021 Jan 18;11:63. doi: 10.1038/s41398-020-01186-6 (PMC7813870; doi:10.1038/s41398-020-01186-6)
Supplement: Supplementary file 1 — Suppl Fig Table Legends [file 41398_2020_1186_MOESM1_ESM.docx]

**Supplementary Information**

**Supplementary Figure 1.** (A) Study design. (B) A matrix of correlation coefficients and scatterplots of DNA methylation showing the relationship between samples for comparisons of the hippocampus (HPC) in female offspring born to mothers that received synthetic glucocorticoids (HPC_S: n=6) compared to saline condition (HPC_C: n=6) and (C) for the comparisons of DNA methylation in whole blood in female offspring born to mothers that received synthetic glucocorticoids (BL_S: n=6) compared to saline controls (BL_C: n=6).

**Supplementary Table 1.** Full list of differentially methylated CpG sites for (A) the hippocampus, (B) for whole blood and (C) those common between the two tissue types at the same CpG sites. (D) Full list of genes containing DMCs in both the hippocampus and blood.

**Supplementary Table 2:** Full list of Gene Ontology terms of the genes differentially methylated in both the hippocampus and whole blood.
