# Supplementary figures and images for: DNA methylome signatures of prenatal exposure to synthetic glucocorticoids in hippocampus and peripheral whole blood of female guinea pigs in early life
Source: Transl Psychiatry. 2021 Jan 18;11:63. doi: 10.1038/s41398-020-01186-6 (PMC7813870; doi:10.1038/s41398-020-01186-6)

## Supplementary Figure 1

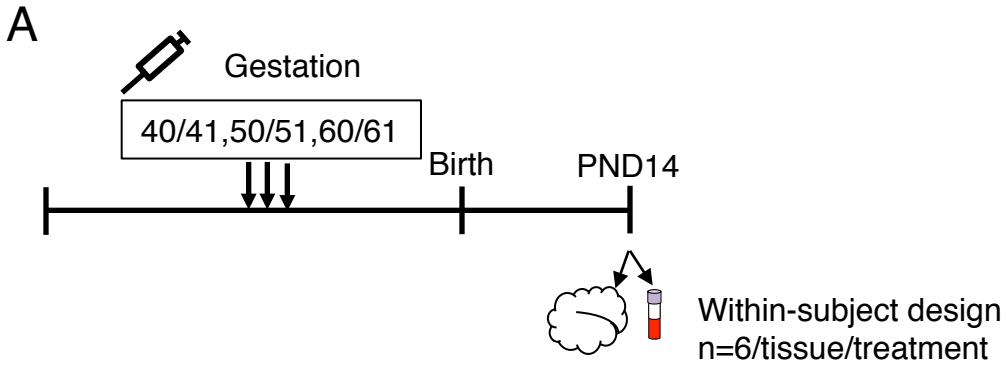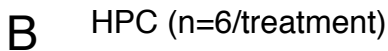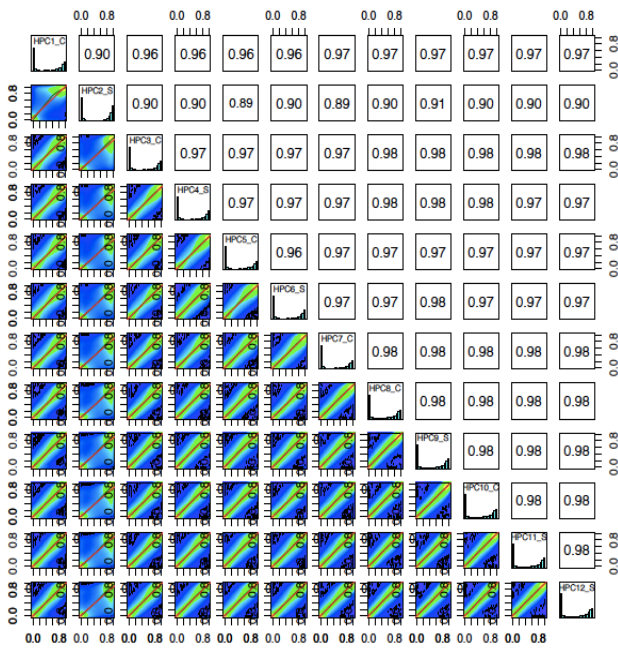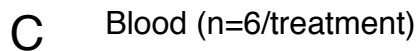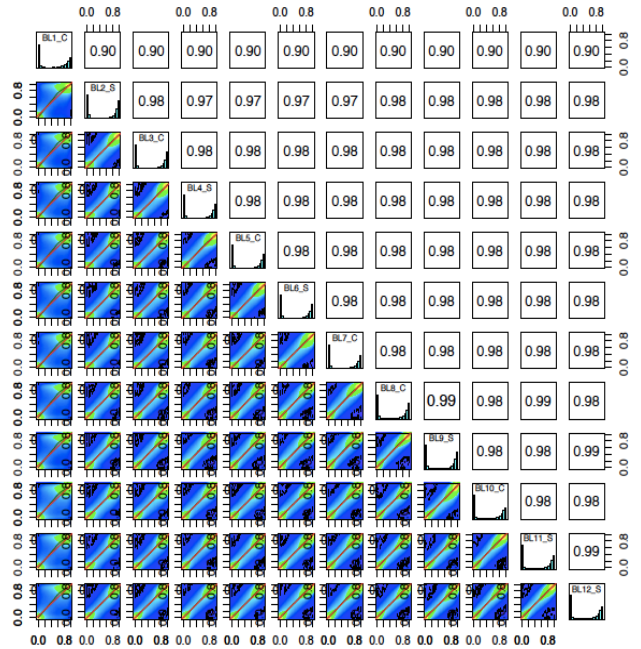

Supplement: Supplementary file 2 — Suppl Fig1 [file 41398_2020_1186_MOESM2_ESM.pdf]
